# Supplementary material for: Fluctuating environments select for short-term phenotypic variation leading to long-term exploration
Source: PLoS Comput Biol. 2019 Apr 19;15(4):e1006445. doi: 10.1371/journal.pcbi.1006445 (PMC6474582; doi:10.1371/journal.pcbi.1006445)
Supplement: S1 Appendix — Comparison of the single-step mutational landscape with a mutational survey incorporating all mutational types. We show that they are qualitatively similar. (PDF) [file pcbi.1006445.s001.pdf]

Fluctuating environments select for short-term phenotypic variation leading to long-term exploration: Supplemental Materials

Rosangela Canino-Koning[1,2*], Michael J. Wiser[2,3], Charles Ofria[1,2,3]

1 Department of Computer Science and Engineering, Michigan State University, East Lansing, MI, USA
2 BEACON Center for the Study of Evolution in Action, Michigan State University, East Lansing, MI, USA
3 Ecology, Evolutionary Biology, and Behavior, Michigan State University, East Lansing, MI, USA

* caninoko@msu.edu

S1 Appendix

Sampled Nearby Mutational Landscape

As noted in equations 1 through 3, the mutants in the nearby mutational landscape include those that have more than one mutation. However, for completeness, we performed an exhaustive landscaping of the single-step mutational landscape, which, by definition, only includes mutants with a single mutation (see Figure S1). In order to verify that our results are indeed representative of the expected genomic and phenotypic diffusion rates, we sampled the mutants in the nearby mutational landscape using all naturally occurring mutations, including multiple mutations in a single mutant.

$$\mu_{pheno} = \mu(1 - p_{p\nu}) \tag{1}$$

$$F_{p\nu} = (1 - \mu_{pheno})^l \tag{2}$$

$$D_p = F_\nu - F_{p\nu} \tag{3}$$

Our results (see figure S1) were virtually identical, showing that the sampling approach and the exhaustive landscaping produce qualitatively indistinguishable results.

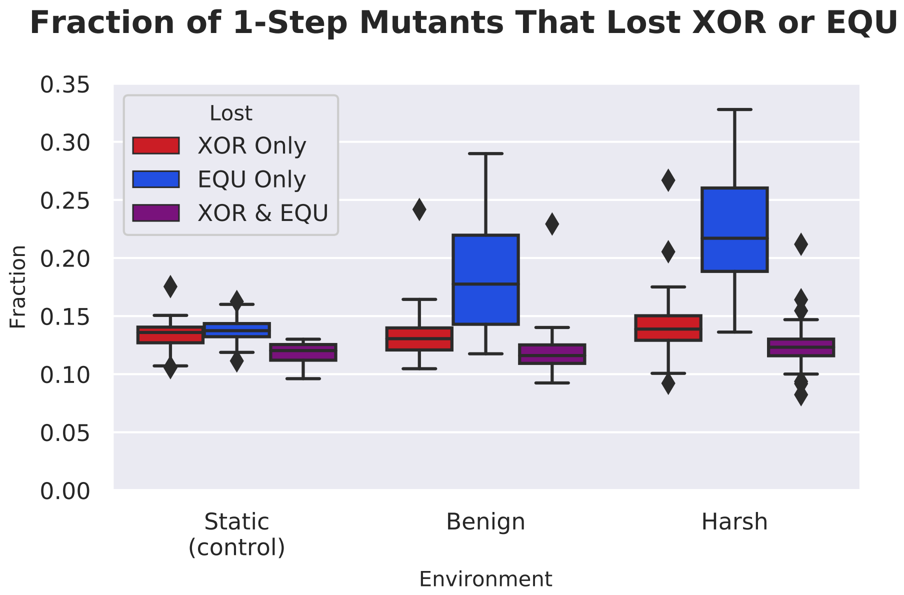

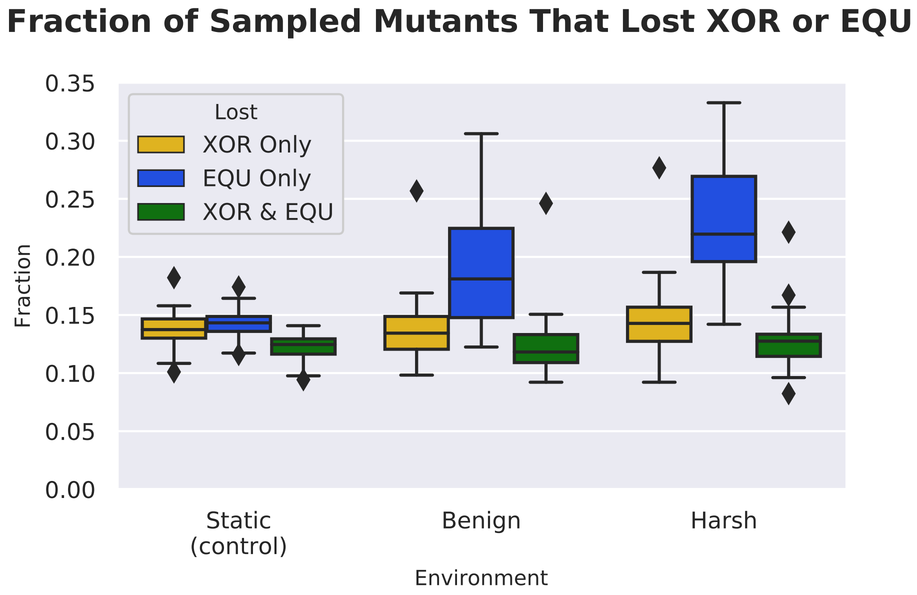

Fig S1. A survey of the single-step and sampled mutational neighborhoods around organisms that performed the fluctuating task. The results are qualitatively identical to each other.
